# Effect of a Multistage Educational Skill-Based Program on Nurse's Stress and Anxiety in the Intensive Care Setting: A Randomized Controlled Trial

**DOI:** 10.1155/2021/8811347

**Published:** 2021-04-26

**Authors:** Mohsen Saffari, Farshid Rahimi Bashar, Amir Vahedian-Azimi, Mohamad Amin Pourhoseingholi, Leila Karimi, Morteza Shamsizadeh, Keivan Gohari-moghadam, Amirhossein Sahebkar

**Affiliations:** ^1^Health Research Center, Life Style Institute, Baqiyatallah University of Medical Sciences, Tehran, Iran; ^2^Health Education Department, Faculty of Health, Baqiyatallah University of Medical Sciences, Tehran, Iran; ^3^Department of Anesthesiology and Critical Care, School of Medicine, Hamadan University of Medical Sciences, Hamadan, Iran; ^4^Trauma Research Center, Nursing Faculty, Baqiyatallah University of Medical Sciences, Tehran, Iran; ^5^Gastroenterology and Liver Diseases Research Center, Research Institute for Gastroenterology and Liver Diseases, Shahid Beheshti University of Medical Sciences, Tehran, Iran; ^6^Behavioral Sciences Research Center, Life Style Institute, Nursing Faculty, Baqiyatallah University of Medical Sciences, Tehran, Iran; ^7^Chronic Diseases (Home Care) Research Center, Hamadan University of Medical Sciences, Hamadan, Iran; ^8^Medical ICU and Pulmonary Unit, Shariati Hospital, Tehran University of Medical Sciences, Tehran, Iran; ^9^Applied Biomedical Research Center, Mashhad University of Medical Sciences, Mashhad, Iran; ^10^Biotechnology Research Center, Pharmaceutical Technology Institute, Mashhad University of Medical Sciences, Mashhad, Iran; ^11^School of Pharmacy, Mashhad University of Medical Sciences, Mashhad, Iran

## Abstract

**Background:**

Psychological problems such as stress and anxiety are prevalent among working nurses in the intensive care units (ICUs). This study was aimed at investigating the effects of three skill-based educational programs on stress and anxiety among critical care nurses.

**Methods:**

Using a randomized controlled trial, 160 nurses were assigned to four groups including one control and three intervention groups. A standard skill-based educational program was delivered to three intervention groups using booklet, booklet+oral presentation, and booklet+oral presentation+clinical teaching over a period of one month to reduce different types of stress and anxiety. The control group received routine education only. Perceived stress, state anxiety, trait anxiety, and work-related stress were assessed at baseline and three times after the intervention (15 days, 3 months, and 21 months). Repeated-measure analysis of variance was used for data analysis.

**Results:**

There was no significant change in the control group in terms of study variables during follow-up assessments, whereas measures of stress and anxiety were reduced after intervention in the trial groups except trait anxiety. Nurses in the mixed-method group (booklet+oral presentation+clinical teaching) showed less stress and anxiety during follow-ups. Although the stress and anxiety scores decreased in the first and second follow-ups, there was no significant reduction in the third follow-up.

**Conclusions:**

To improve the mental health and performance of the intensive care unit nurses, knowledge-based and skill-based training programs seem useful. Continuous training may help to maintain the effectiveness of these programs over time.

## 1. Introduction

Stress and anxiety may be used interchangeably but have different definitions. Stress has been defined as a mental, physical, or emotional strain perceived to be beyond individual's resources, whereas anxiety is an emotion specified by feelings of tension, worry, intrusive thought, and physiological changes such as high blood pressure and heart rate. Therefore, anxiety may be considered as a reaction to a stressful event [1]. This event may be related to the work environment. In fact, nursing, teaching, and management may produce more stress and anxiety for their practitioners than other jobs [2, 3].

Nursing staff working in healthcare settings regularly experience high mental and physical distress that may affect their quality of life and health status [4]. Nurses as a large part of the healthcare personnel not only provide patient care support but also contribute to rehabilitation programs, provide emotional support for patients and their families, and participate in promoting health throughout the community [5]. Other roles such as patient education, communication with other members of healthcare team (including physicians, psychologists, and social workers), and providing adequate prevention services to healthy people may also be attributable to nurses [6]. So, this multifaceted structure of workload is a key job stressor for nurses. Nurses are at higher risks for developing mental health problems such as stress and anxiety than other healthcare professionals and the general population [7–9]. Studies indicate a global prevalence rate of stress and anxiety disorders ranging from 20% to 66% among this population [3, 10]. The problem seems to be more drastic in developing countries with serious nurse shortages. Also, there is evidence that about half of the Iranian nurses is exposed to severe stressful events over their work time [11, 12].

Nurses who work in intensive care units (ICUs) often encounter emergencies and critical situations more frequently than those from other wards [11]. A study conducted in Sweden showed that more than two-thirds of nurses working in ICUs reported higher levels of psychological distress than their counterparts in other wards [13]. Indeed, the ICU is a demanding, fast-paced, and tension-arousing environment that predisposes nurses to high stress and anxiety through factors such as long shift work, exposure to severe diseases as well as deaths, excessive work load, negative emotions, and limited authority [14, 15]. Also, other individual factors including sleep deprivation and fatigue which are common in ICU nursing staff may worsen the situation. Prolonged exposure to such factors may lead to work-related burnout and develop higher risks of morbidities such as metabolic disorders, coronary heart diseases, and autoimmune conditions [16]. Moreover, poor mental health caused by stress and anxiety may affect decision-making capability and increases medical care errors that finally will be conducive to threaten patients' health [17]. Studies also show some nurses may be satisfied with working in the ICUs and many nurses may be affected by psychological distresses resulting in sickness and absence [14, 18]. Therefore, ICUs are usually vulnerable to high turnover and nursing shortages [1].

Despite significant negative effects of stress and anxiety on nurses especially in ICUs, there are few effective strategies to tackle and prevent these problems. Over the past two decades, several interventions have been proposed to assist nurses in managing their stress and anxiety [19]. Majority of these interventions are psychological-based. Use of individualized stress management programs, training relaxation techniques as well as mindfulness and meditation, and using humor, cognitive modifications, and self-relaxation biofeedback are some examples of such interventions with different effectiveness [20].

Insufficient skill and information along with professional inability are some of the most important sources of stress and anxiety for ICU nurses [19]. Although nurses who graduated from nursing colleges or universities are expected to be adequately trained to perform nursing tasks, working in high demand wards such as ICUs needs special skills and competencies, and even experienced nursing staff should keep themselves updated with the latest practice guidelines periodically [21].

There are some studies which addressed the necessity of skill-based educations for ICU nurses. Jansson et al. in a systematic review attempted to assess the effect of simulation-based education for critical care nurses. They found only one qualified study that showed this kind of education may improve nurses' adherence regarding medication usage, patient safety, and providing a qualified care for patients [22]. In another study, De Silva et al. designed and evaluated a skill-training program using both local and overseas nurse trainers. They found that nurses who took part in the course had significantly higher knowledge and skills compared to controls in terms of clinical assessment and providing healthcare procedures [23]. Another study showed that a program to improve delirium-screening skill among ICU nurses improved either knowledge or skills of the participants after 10 months of education [24]. Although such studies may confirm the effectiveness of education programs conducted in intensive care settings to enhance knowledge and skills of healthcare workers, there is no clear evidence on how such education may affect the mental health of nurses. Therefore, the current study was performed to investigate the effects of ICU nurses' training using a skill-based education program in the form of some progressive steps on their stress and anxiety.

## 2. Methods

### 2.1. Study Design

We conducted a prospective, randomized, parallel, controlled triple-blinded trial with repeated measurements. The reporting of this study complies with the CONSORT (Consolidated Standards of Reporting Trials) statement for trials of nonpharmacological treatments [25]. The trial was registered with ClinicalTrials.gov (identifier NCT02838160). The protocol is available for review upon reasonable request. All nurses, data collectors, and the statistician were blinded to group assignment.

### 2.2. Participant and Setting

The multicenter study was conducted in four academic teaching hospitals in four mixed medical surgical intensive care units (ICU) in Tehran, Iran. The study consisted of a 3-month baseline observation period (October 2011–December 2011, Period 1), followed by a 6-month intervention period (January 2012 to June 2012) and 3-month (July 2012–September 2012, Period 2), 15-month (October 2012–December 2013, Period 3), and 21-month (January 2014–June 2015, Period 4) follow-up periods. The study protocol remained unchanged throughout the study period in all ICUs. Inclusion criteria were holding a degree qualification as a registered nurse and being a direct care provider (bedside). Nurses with less than one-year experience in the critical care unit or working less than the whole study period were excluded.

### 2.3. Randomization

**Figure 1:**
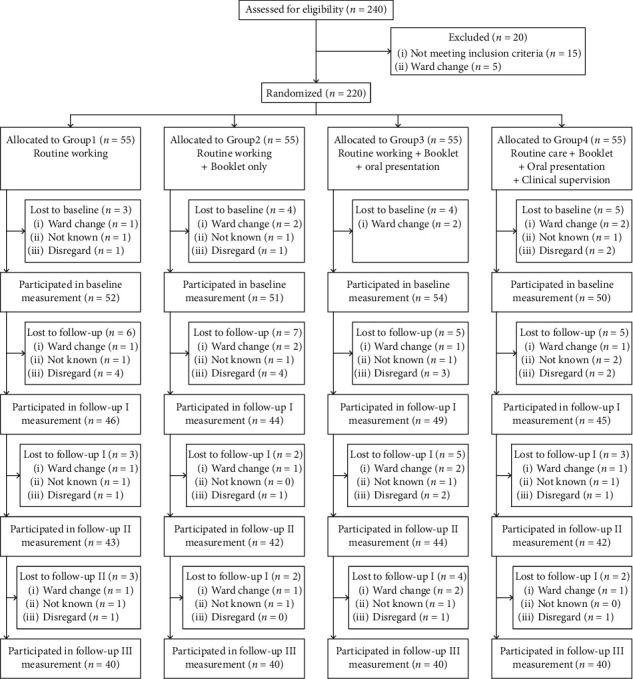
Flow diagram of nurse's enrollment.

Convenience sample of critical care nurses were recruited through letters and telephone and face*-*to*-*face invitations. Included nurses were randomly assigned to control or one of the three competing intervention groups (allocation ratio of 1 : 4). Block randomization (groups of 4) was performed using a random number list generated by Random Allocation Software© (RAS; Informer Technologies, Inc., Madrid, Spain; Figure 1). Numbers were placed into sequential containers that were kept in a secure location until allocation consignment. To ensure blinding, the allocation sequence was kept by a different investigator. A third investigator was responsible for nurse follow-up and assessments. There were no important changes to the methods after trial commencement.

### 2.4. Ethical Considerations

Before the trial started, the protocol was approved by the investigational review board at the participating medical center in Hamadan, Iran. Written informed consent was obtained in the study. Consent covered both study participation and consent to data publication.

### 2.5. Availability of Data and Materials

All relevant data are within the paper and its supporting information files. Deidentified individual subject data may be available from the corresponding author on reasonable request.

### 2.6. Intervention

A predefined ventilator bundle was designed and validated using the Delphi technique in three rounds to test concordance using Cohen's kappa coefficient (*κ*) in a multidisciplinary research team. This team was comprised of 21 experts, who were selected for their methodological and/or clinical ICU expertise. The predefined ventilator bundle contains 17 components divided to six main categories: (1) interventions to prevent complications associated with mechanical ventilation (e.g., peptic ulcer disease and deep venous thrombosis prophylaxis), (2) interventions to reduce the duration of sedation (e.g., daily sedation interruption), (3) interventions to reduce the duration of mechanical ventilation (e.g., spontaneous breathing trial), (4) interventions to prevent the risk of aspiration of oropharyngeal and/or gastric content (e.g., elevation of the head of the bed, subglottic suctioning, and cuff pressure control), (5) interventions to reduce the microbiological colonization of the airways (e.g., daily oral care with chlorhexidine), and (6) interventions to prevent person-to-person transmission of bacterial infection (e.g., strict hand hygiene and personal protective equipment) [26–28]. Consensus between the research team was high (*κ* = 0.943).

The research team designed a 15-page self-study booklet and held a series of 90–120-minute oral presentations covering the scope (e.g., definition, epidemiology and etiology, risk factors, and clinical and economic outcomes) and management of the problem (i.e., 17 ventilator bundle components). After baseline measurement, Group 1 did not receive any intervention or education. Group 2 received only the designed booklet without any participation in the oral presentation. Group 3 participated in oral presentations 14 days after completing the self-study booklet. Interactive, standardized, and mandatory oral presentations were held five times to ensure maximum attendance of the three nursing shifts. Respectively, Group 4 received clinical teaching in the bedside after completing the self-study booklet and a series of interactive, standardized, and mandatory 90–120-minute oral presentations. During the clinical teaching, participants' compliance with the ventilator bundle was ensured by the members of the research team. Contrary to the intervention groups (Groups 2–4), the control group (Group 1) did not receive any educational intervention during the study period. For observing the ethical consideration, the members in Group 1 received booklet education after follow-ups and when the data collection was finished.

### 2.7. Data Collection

The primary outcome was the nurse's stress and the secondary was the nurse's anxiety. For data collection, several tools were used.

### 2.8. Demographic Variables

Recorded data included age, gender, qualifications, marital status, working shift, number of children, working in holiday, clinical experiences, collaboration, supportive supervisor, and clinical competency. Collaboration means “a complex process in ICU nursing which is important to do procedures, educations, and ICU related matters, efficiently. In other words, the variable measures the harmonization, unification, cooperation, coordination, and sense of working together level in an environment with critically ill patients and stressful situation.” Supportive supervision means “the existence of supportive supervisor from a higher level observing the team performance in ICU.” Clinical competency means “the level of working nurses in ICU to do the clinical practices according to the latest guideline and his/her skill in doing the procedures.” For spectral variables (collaboration, supportive supervisor, and clinical competency), items were scored by three colleagues of the nurse from 0 to 15. Nurses were trained for scoring variables. Three spectral variables were scored independently of other scores. The scoring was done eight times, at the beginning of each three-month period; the total score was the mean value of the 8 measures. The process was done independently for each spectral variable. The Kendal coefficient correlation between three scorers was *r* = 0.91 with *P* < 0.0001.

### 2.9. Perceived Stress Questionnaire (PSQ-14)

PSQ-14 scores are obtained by reversing the scores on the five positive items, including 0 = 4, 1 = 3, 2 = 2, 3 = 1, and 4 = 0, then summing across all 14 items. Scores range from 0 to 70 [29]. The reliability of the PSQ-14 was assessed in this study by test-retest and Cronbach's alpha, 0.93 and 0.92, respectively. The scale has demonstrated solid psychometric properties among the Iranian population [30].

### 2.10. State-and-Trait Anxiety Questionnaire (STAQ)

This validated tool has 20 items for assessing trait anxiety and state anxiety [31]. All items are rated on a 4-point scale ranging from very low (1 point) to very high (4 points). Scores range from 20 to 80. Higher scores indicate greater anxiety. The reliability of the state-trait anxiety questionnaire was assessed in this study by test-retest and Cronbach's alpha, 0.89 and 0.90, respectively. This scale has been previously validated for Iranian people [32].

### 2.11. Nurses' Critical Care Stressor Questionnaire

Working nurses' stressors in critical care units was developed by Vahedian-Azimi et al. [1, 11]. Items focused on different stressors in real situations of ICUs. The respondents were asked to rate the work stressors on a five-point Likert scale ranging from “causes me no stress” to “causes me extreme stress” with this explanation “please tell us the degree of being stressors of each question.” The minimum and maximum scores of the instrument were 22 and 110, respectively. The reliability of the questionnaire was assessed in this study by test-retest and Cronbach's alpha, 0.91 and 0.92, respectively. This scale showed acceptable results in terms of validity assessment in a previous study among nurses [1, 11].

### 2.12. Sample Size and Data Analysis

The sample size was determined through power analysis, which revealed that a sample size of 40 was required to detect a 20% difference between state anxiety rates in each group (*α* = 0.05, 1 − *β* = 0.9; dropout = 20%). The estimation of the effect size is based on a previous pilot study, conducted from March 2010 to December 2010 (unpublished data). In this pilot study, with 10 eligible persons in each group and after about 8 weeks, the primary results were analyzed for stress and anxiety, and the minimum differences across groups were used in the sample size calculation.

Discrete variables are expressed as counts and percentages, and continuous variables are expressed as means and standard deviations (SDs). To compare numerical variables, analysis of variance (ANOVA), and for repeated factors in follow-up, repeated-measure analysis (adjusted for baseline values as covariates), were used. Bonferroni correction was used for post hoc multiple comparisons. For categorical data, chi-square test or Fisher's exact test was employed, as appropriate. All *P* values less than 0.05 were considered statistically significant.

## 3. Results

### 3.1. Nurse Characteristics

The total of 160 ICU nursing staff participated, 71.9% of nurses were women, and the mean ± SD age of nurses was 41.11 ± 5.93 years. About 51.9% had secondary education, 60.6% were married, and mean years of ICU experience were 18.11 ± 6.18. No demographic variable differed among the groups (Table 1).

### 3.2. Perceived Stress

According to repeated-measure analysis (controlling for baseline values as covariates), both between- and within-group effects appeared significant, and the interaction between time and treatments was also significant, indicating that the implementation of the educational program led to reduction in the perceived stress, compared to controls during the follow-up (Table 2).

**Figure 2:**
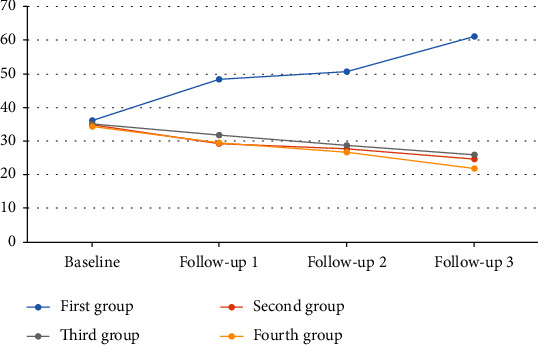
The trend of perceived stress from baseline up to 21-month follow-up.

Bonferroni's post hoc analysis showed significant differences between routine care (controls) and other treatment groups (*P* < 0.001). Moreover, routine care+booklet+oral presentation+clinical supervision indicated a better decreasing effect on stress compared to routine care+booklet+oral presentation (*P* = 0.003). In general, perceived stress was reduced in treatment groups during the follow-up (Figure 2).

### 3.3. State Anxiety

According to repeated-measure analysis (controlling for baseline values as covariates), both between- and within-group effects were significant, and the interaction between time and treatments was also significant, indicating that the implementation of the educational program led to reduction in the state anxiety for treatments compared to controls, during the follow-up (Table 3).

**Figure 3:**
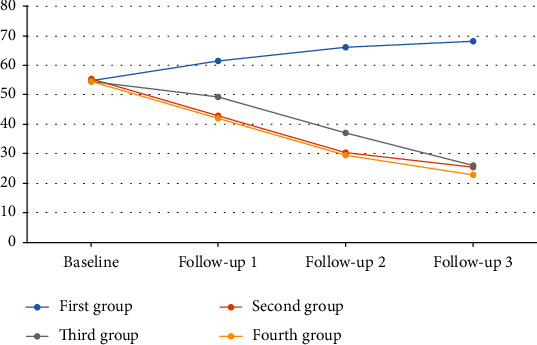
The trend of state anxiety from baseline up to 21-month follow-up.

Bonferroni's post hoc analysis showed significant differences between routine care (controls) and other treatment groups (*P* < 0.001). Post hoc analysis also indicated that both routine care+booklet only and routine care+booklet+oral presentation+clinical supervision were associated with stronger reductions in state anxiety, compared to routine care+booklet+oral presentation (*P* < 0.001). In general, state anxiety was reduced in the treatment groups during the follow-up (Figure 3).

### 3.4. Trait Anxiety

**Figure 4:**
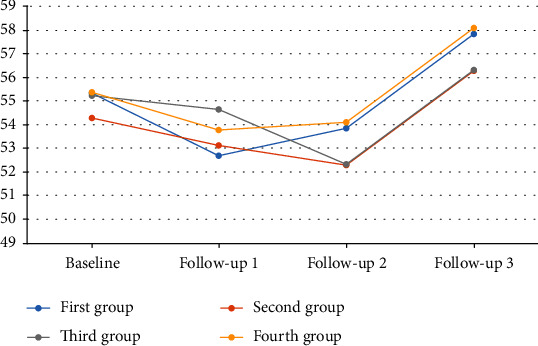
The trend of trait anxiety from baseline up to 21-month follow-up.

According to repeated-measure analysis (controlling for baseline values as covariates), there were no statistical differences across groups with respect to decrease in trait anxiety, and no statistically significant difference was observed between treatment and control groups over the follow-up period (Table 4). There was no significant trend for trait anxiety during the follow-up (Figure 4).

### 3.5. ICU Working Stress

According to repeated-measure analysis (controlling for baseline values as covariates), both between- and within-group effects were significant, and the interaction between time and treatments was also significant, indicating that the implementation of the educational program led to the reduction of the ICU working stress for the treatment groups compared to controls, during the follow-up (Table 5).

**Figure 5:**
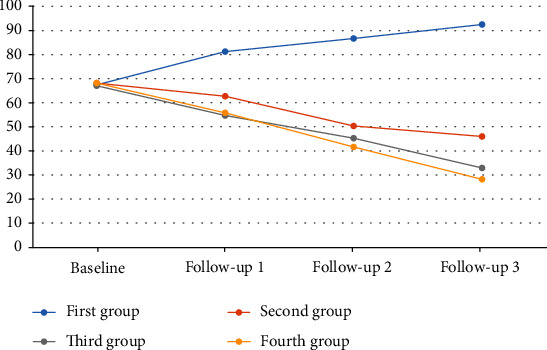
The trend of ICU working stress from baseline up to 21-month follow-up.

Bonferroni's post hoc analysis showed significant differences between routine care (controls) and other treatment groups (*P* < 0.001). Post hoc analysis also indicated that subjects in the routine care+booklet+oral presentation+clinical supervision group experienced more ICU working stress reduction compared to the other treatment groups (*P* < 0.001). In general, working stress was reduced in the treatment groups during the follow-up (Figure 5).

## 4. Discussion

The present study investigated the effect of an education program to reduce stress and anxiety among ICU nursing staff. The findings supported the effectiveness of the program to reduce perceived stress, state anxiety, and ICU working stress but not trait anxiety. Also, we found that educational content delivered through various methods may increase effectiveness of education to improve the psychological status especially for stressors related to the work setting. However, the descending impact of the program over time makes necessary the replication of such programs via in-service training schedules.

These findings were consistent with previous studies which evaluated the effectiveness of education programs on stress and anxiety among nurses. For example, Moayed et al. set an education on stress of exposure to sharps for nurses in an emergency setting and found that this type of education may reduce the stress related to needle stick injuries among the participants significantly [33]. The method of education was oral as in the present study and reduced stress among nurses, although these observations were based on a quasiexperimental design with only 35 nurses followed for one month. A recent study in Iran also revealed that education programs may help to reduce stress in nurses employed in the neonatal ICUs. In this study, 70 nurses were assigned to intervention and control groups, and the McNamara education program, complemented by lecture-based presentations, was chosen as a basis for intervention in the experimental group. Two weeks after the intervention, the experimental group showed lower scores on a stress response inventory than controls, thus supporting effectiveness of the program [34].

A few studies assessed the effectiveness of education programs on anxiety among nurses that generally have emphasis on cognitive-based interventions. Ghazavi et al. studied how a happiness-based educational program may be effective to reduce stress and anxiety in cancer patients' nurses. They recruited 52 nurses and using a RCT design assigned them to intervention and control groups. The education program was conducted over a six-week course and utilized lectures and discussions to educate participants regarding happiness. They measured the outcomes two times, immediately after the intervention and one month later. The findings showed that at both follow-ups, the stress and anxiety levels in the intervention group were lower than those in the control group. Moreover, as in the present study, the impact of such program decreased over time [35]. Another pilot study conducted on 20 nurses and midwives using a one-day workshop, a mindfulness-based stress reduction program, did not find significant changes in scores on depression, anxiety, and stress scale (DASS) after two months [36]. This study along with other similar studies [37–39] suggested that a comprehensive and multifaceted intervention could reduce the anxiety level as well as our intervention.

An interesting finding of our study was that education on professional skills in nurses did not have significant impact on trait anxiety. Based on definition, trait anxiety is related to experience and reports negative feelings and emotions such as fears and concerns across different conditions. Indeed, this is a personality aspect that may be manifested in those with neurotic disorders. Also, those who have a stable perception regarding threatening environmental factors may demonstrate a considerable trait anxiety. On the other hand, state anxiety is an undesirable emotional arousal from exposure to threatening situations or demand that may be decreased by removing these situations [40].

Therefore, because our intervention focused on improving professional knowledge and skills of the nurses for enhancing ICU performance, it may generally address gaps or deficits in the expertise of nurses and help them to manage demanding or unpleasant situations, whereas changing negative feelings or emotions encompassed in trait anxiety could not be achieved by a professional skill-based program. Trait anxiety is a personality component of one's psychological status that could not be easily changed by educational interventions.

The current study suggested the necessity of ongoing similar programs to maintain the effectiveness of educational intervention. Our three follow-ups performed at 3, 15, and 21 months after the intervention showed that stress and anxiety scores moderately increased over time. Therefore, consistent with previous studies [41–43], the effect of education may fade out over time and needs to be strengthened by periodic educational programs or planning in-service trainings on professional expertise.

The present study includes some limitations that should be considered. First, we only addressed the nurses working in the ICUs; thus, our results could not be generalized to other wards or settings. Second, we only focused on the education of skills and professional knowledge necessary for working in the ICUs, and we did not consider other types of interventions with emphasis on cognitive therapy or mindfulness that generally are used as stress management techniques. Therefore, future studies might assess the impact of a combination of such approaches on nurses' stress and anxiety. Third, there are other psychological outcomes such as self-confidence and self-efficacy that may be affected by such an educational program, but due to some limitations regarding the study time and data collection process, we decided to skip such outcomes in our study that may be examined in the future similar studies. Fourth, along with ICU nurses, other healthcare professionals including auxiliary healthcare personnel and physicians may also have experienced a considerable amount of stress and anxiety which has not been addressed in this study. Finally, as the total study period was approximated two years, it should be noted that the participants were not involved in other educational training programs that could have improved their knowledge and skills and, subsequently, reduced their anxiety and stress. Nevertheless, lapse of time may have a maturation effect on the participants' knowledge and skills, but since this effect is expected to occur in all four groups, the potential confounding effect might be considered as minimal.

## 5. Conclusions

In summary, the current study showed that a knowledge- and skill-oriented education program may help the ICU nurses to overcome their stress and anxiety in the work setting. This finding suggested that a notable part of mental health problems including job stress and anxiety among nurses may be prevented by providing and refreshing knowledge and skills needed for work in ICUs. Therefore, planning in-service training especially for such nurses might improve the quality of services provided by the nurses and also contribute to reduce stress and anxiety in this population. Designing similar interventions for use among other healthcare professionals as well as considering other psychological factors which may be under the influence of such programs (e.g., self-efficacy and self-confidence) may help decision makers and healthcare authorities to integrate such interventions for continuing education programs in nurses and other members of the healthcare team.

## Figures and Tables

**Table 1:** Demographic variables of nurses in 4 educational groups (*n* = 55 nurses per group).

Variables	Groups				Total	*P* value
	Routine working	Routine working+booklet	Routine working+booklet+oral presentation	Routine working+booklet+oral presentation+clinical supervision		
Age, mean (SD)	40.77 (6.35)	42.02 (6.23)	41.27 (5.56)	40.37 (5.65)	41.11 (5.94)	0.636
Gender, female (%)	29 (72.5)	30 (75)	29 (72.5)	27 (67.5)	115 (71.9)	0.899
Education degree, baccalaureate (%)	22 (55)	18 (45)	19 (47.5)	24 (60)	83 (51.9)	0.345
Marital status, married (%)	25 (62.5)	22 (55)	27 (67.5)	23 (57.5)	97 (60.6)	0.315
Working shift, rotation (%)	8 (20)	14 (35)	12 (30)	9 (22.5)	43 (26.9)	0.282
Number of children, two (%)	18 (45)	20 (50)	13 (32.5)	10 (25)	61 (38.1)	0.991
Working in holiday, yes (%)	23 (57.5)	24 (60)	23 (57.5)	24 (60)	94 (58.8)	0.154
Collaboration, median (IQR)	3 (3–4)	3 (3–4)	3 (3–4)	3 (3–4)	3 (3–4)	0.308
Supportive supervisor, median (IQR)	3 (2–4)	3 (2–4)	3 (2–4)	3 (3–4)	3 (2–4)	0.821
Clinical experiences, mean (SD)	18.10 (6.39)	18.65 (6.65)	18.20 (6.69)	17.47 (5.01)	18.11 (6.18)	0.867
Median (IQR)	18 (13–24)	20 (13–24)	20 (13–23)	18 (13.25–21.50)	18 (13–23)	
Clinical competency, mean (SD)	12.32 (1.66)	12.27 (1.74)	12.62 (1.29)	12.40 (1.19)	12.41 (1.48)	0.731
Median (IQR)	12 (11–14)	12 (11–14)	13 12–14)	12 (12–13)	12 (11–14)	

**Table 2:** Comparisons between trial and control groups in terms of perceived stress at baseline (time 1), 6 months (time 2), 15 months (time 3), and 21 months (time 4) after the intervention.

Time	Time 1	Time 2	Time 3	Time 4	Between-group comparison (treatments)	Within-group comparison (time)	Interaction between time and treatments
Group	M (SD)	M (SD)	M (SD)	M (SD)			
Control	36.05 (3.11)	48.37 (3.69)	50.65 (3.75)	61.1 (4.06)	*P* < 0.001	*P* < 0.001	*P* < 0.001
Inter. 1	34.78 (2.91)	29.35 (4.38)	27.6 (4.56)	24.53 (4.44)			
Inter. 2	35.03 (2.99)	31.87 (3.24)	28.8 (3.42)	25.85 (3.86)			
Inter. 3	34.45 (3.19)	29.6 (3.46)	26.65 (3.85)	21.73 (4.35)			

M: mean; SD: standard deviation; NS: nonsignificant; inter.: intervention.

**Table 3:** Comparisons between trial and control groups in terms of state anxiety at baseline (time 1), 6 months (time 2), 15 months (time 3), and 21 months (time 4) after the intervention.

Time	Time 1	Time 2	Time 3	Time 4	Between-group comparison (treatments)	Within-group comparison (time)	Interaction between time and treatments
Group	M (SD)	M (SD)	M (SD)	M (SD)			
Control	54.68 (3.37)	61.55 (3.35)	66.03 (2.42)	68.18 (1.57)	*P* < 0.001	*P* < 0.001	*P* < 0.001
Inter. 1	55.38 (2.98)	42.98 (3.35)	30.35 (3.79)	25.3 (3.63)			
Inter. 2	54.63 (2.88)	49.28 (3.08)	37.0 (3.57)	26.08 (3.68)			
Inter. 3	54.35 (2.81)	41.95 (2.91)	29.63 (3.51)	22.93 (2.72)			

M: mean; SD: standard deviation; NS: nonsignificant; inter.: intervention.

**Table 4:** Comparisons between trial and control groups in terms of trait anxiety at baseline (time 1), 6 months (time 2), 15 months (time 3), and 21 months (time 4) after the intervention.

Time	Time 1	Time 2	Time 3	Time 4	Between-group comparison (treatments)	Within-group comparison (time)	Interaction between time and treatments
Group	M (SD)	M (SD)	M (SD)	M (SD)			
Control	55.35 (4.91)	52.68 (4.69)	53.85 (4.02)	57.85 (4.03)	0.233 NS	0.670 NS	0.769 NS
Inter. 1	54.3 (4.81)	53.13 (4.07)	52.3 (4.64)	56.3 (4.64)			
Inter. 2	55.23 (4.16)	54.65 (3.87)	52.33 (4.40)	56.33 (4.40)			
Inter. 3	55.38 (4.48)	53.78 (4.25)	54.1 (3.47)	58.1 (3.47)			

M: mean; SD: standard deviation; NS: nonsignificant; inter.: intervention.

**Table 5:** Comparisons between trial and control groups in terms of ICU working stress at baseline (time 1), 6 months (time 2), 15 months (time 3), and 21 months (time 4) after the intervention.

Time	Time 1	Time 2	Time 3	Time 4	Between-group comparison (treatments)	Within-group comparison (time)	Interaction between time and treatments
Group	M (SD)	M (SD)	M (SD)	M (SD)			
Control	67.25 (2.85)	81.00 (3.58)	86.68 (3.88)	92.45 (4.62)	*P* < 0.001	*P* < 0.001	*P* < 0.001
Inter. 1	68.28 (2.79)	62.7 (3.24)	50.3 (3.24)	45.98 (3.22)			
Inter. 2	67.15 (2.33)	54.75 (2.36)	45.15 (3.25)	32.83 (3.67)			
Inter. 3	67.93 (3.30)	55.6 (3.61)	41.55 (4.09)	28.13 (3.63)			

M: mean; SD: standard deviation; NS: nonsignificant; inter.: intervention.

## Data Availability

Data are available from the first and corresponding authors upon reasonable request.
